# Supplementary material for: Bunyaviruses Affect Growth, Sporulation, and Elicitin Production in Phytophthora cactorum
Source: Viruses. 2022 Nov 22;14(12):2596. doi: 10.3390/v14122596 (PMC9788385; doi:10.3390/v14122596)
Supplement: Supplementary file 1 [file viruses-14-02596-s001.zip › Supplementary_Table S1.pdf]

			Read counts PhF66- (no viruses)					PhF66 (PcBV1&PcBV2)							
**Accession**	**Description**	**length**	**R1**	**R2**	**R3**	**R7**	**R8**	**R4**	**R5**	**R6**	**R9**	**R10**	**log2Fold Change**	**FDR**	**Padj**
KAF1787014.1	hypothetical protein GQ600_18332	581	0	0	0	0	0	12,91	17,78	21,27	21,19	15,59	6,56	0,000002	3.512e-117
KAF1778147.1	Riboflavin synthase-like beta-barrel	1444	1,5	0,92	1,54	4,68	1,66	22,08	54,9	8,28	8,28	10,26	2,70	0,000053	2.961e-25
**KAF1774323.1**	**Elicitin**	**534**	52,63	47,31	47,61	7,9	8,57	45,43	77,86	283,71	293,98	292,02	**2,68**	0	**1.512e-135**
**KAF1774322.1**	**Elicitin**	**516**	0,11	0,16	0	0,08	0	0,07	0,25	2,92	2,77	2,32	**2,49**	0,000513	**1.226e-11**
KAF1789104.1	hypothetical protein GQ600_9925	270	0,75	0,84	0,35	0,45	0,58	4,82	5,01	4,02	2,77	3,87	2,43	0,00005	4.134e-21
KAF1774139.1	hypothetical protein GQ600_10043	470	0,15	0,08	0,04	0,12	0,12	0,91	2,96	0,69	1,26	1,22	2,39	0,000147	2.717e-11
**KAF1781019.1***	**Elicitin**	**357**	1,94	0,72	1,41	0,04	0	0,98	1,34	6,99	28,63	13,27	**2,35**	0,000294	**2.485e-10**
KAF1780261.1	SMP-30/Gluconolactonase/LRE-like region	1114	2,13	2,32	1,19	2,65	1,12	15,59	14,53	8,43	7,82	7,16	2,19	0,000161	1.754e-18
KAF1780813.1	hypothetical protein GQ600_560	264	1,31	1,64	0,84	0,9	0,42	7,4	2,34	7,39	5,93	6,8	2,14	0,000655	3.324e-12
KAF1785474.1	Nucleotide-diphospho-sugar transferase	879	0,82	0,32	0,66	2,28	0,87	6,96	19,45	2,43	2,52	2,77	2,07	0,000655	4.261e-12
KAF1791367.1	hypothetical protein GQ600_10067	351	0,37	1,04	0,71	1,63	0,46	6,67	4,22	3,07	3,91	3,58	1,95	0,000966	1.971e-10
KAF1780813.1	hypothetical protein GQ600_560	587	1,38	0,68	0,88	0,41	1,04	4,68	5,22	3,32	2,9	2,77	1,79	0,000779	2.73e-10
KAF1789026.1	hypothetical protein GQ600_10132	702	0,71	0,56	1,85	3,58	1,04	10,59	16,11	4,41	3,7	3,42	1,79	0,002086	5.979e-09
KAF1779177.1	hypothetical protein GQ600_27577	542	0,04	0,08	0,09	0,04	0	0,94	1,34	0,5	0,55	0,16	1,79	0,004299	6.682e-06
KAF1788372.1	Major facilitator superfamily domain	1378	2,24	3,44	2,47	1,1	0,25	2,32	6,35	7,83	10,22	9,08	1,76	0,001655	7.031e-10
KAF1788051.1	hypothetical protein GQ600_26909	495	0,26	0,24	0,35	12,62	2,08	30,75	66,33	1,74	1,22	1,87	1,74	0,002062	3.684e-06
KAF1782571.1	hypothetical protein GQ600_10993	346	1,57	1,24	1,24	3,22	2,2	12,98	16,07	3,47	2,99	4,03	1,72	0,000287	4.189e-14
KAF1776425.1	hypothetical protein GQ600_14855	296	1,87	2,12	2,47	0,04	0,29	2,43	0,88	7,24	6,39	7,33	1,71	0,003595	1.583e-07
KAF1787242.1	hypothetical protein GQ600_9309	393	0,07	0	0,04	0,49	0,67	1,63	1,88	0,64	0,92	0,57	1,70	0,003778	4.735e-06
KAF1781184.1	**CAP domain**	4548	3,25	4,12	4,9	3,3	3,24	18,64	18,03	9,27	9,5	11,85	1,69	0,000273	2.952e-14
KAF1789435.1	Multicopper oxidase, type 3	2747	1,83	3,76	3,31	0	0	0	0,33	11,75	9,33	12,17	1,67	0,006262	2.229e-08
KAF1788424.1	hypothetical protein GQ600_6414	3948	0,52	0,36	0,26	0,12	0	0,87	0,63	1,54	1,14	1,55	1,65	0,003913	5.982e-06
KAF1783256.1	EGF-like domain	4737	0,67	1,12	0,93	0,29	0	1,34	1	3,12	4,29	1,99	1,65	0,00493	1.281e-06
KAF1785530.1	hypothetical protein GQ600_22799	454	0,15	0,04	0,26	0	0	0,15	0,33	1,54	0,84	0,69	1,64	0,006025	4.821e-05
KAF1780101.1	Amino acid transporter, transmembrane domain	6135	0,71	1,2	0,44	0,29	0,04	0,76	3,13	2,63	2,1	2,85	1,59	0,006382	1.514e-05
KAF1774329.1	**Necrosis inducing protein**	696	4,75	4,44	6,18	0,37	0,17	1,52	4,47	11,55	12,24	13,88	1,57	0,00385	3.247e-07
KAF1789629.1	Zinc finger, C2H2-like	6766	0,11	0,16	0,09	0	0	0,69	2,13	0,89	0,5	0,33	1,57	0,008492	0.0001281
KAF1774124.1	hypothetical protein GQ600_1308	1475	0,37	0,4	0,79	0,08	0,17	1,74	0,79	1,78	1,64	1,3	1,55	0,004658	2.249e-05
KAF1780238.1	Lipoxygenase, C-terminal	1701	12,45	11,56	11,03	5,66	5,66	9,97	18,7	35,94	37,97	36,02	1,54	0,000009	2.814e-29
KAF1795599.1	Hypoxia induced protein, domain	603	5,05	4,6	4,37	0,12	0,29	0,44	0,42	14,33	17,24	13,15	1,54	0,000287	8.566e-14
KAF1775647.1	hypothetical protein GQ600_1350	416	0,04	0	0	0	0	0,04	0	0,64	0,71	0,65	1,54	0,004829	0.0001323
KAF1779203.1	hypothetical protein GQ600_5881	320	0,07	0,04	0,04	4,4	0,42	12,44	16,53	0,55	0,76	0,65	1,53	0,004672	0.0001748
KAF1787920.1	hypothetical protein GQ600_27546	909	1,05	2	1,28	0,65	0,21	2,39	4,93	2,97	3,83	3,62	1,53	0,004782	2.299e-06
KAF1780226.1	hypothetical protein GQ600_22014	557	9,05	7,88	9,49	0,57	0,21	1,23	0,75	26,37	26,78	25,07	1,52	0,000287	3.676e-18

KAF1775607.1*	DNA polymerase family X lyase domain	3771	5,16	5,6	4,77	141,93	116,35	218,74	287,26	20,23	23,5	18,36	1,51	0,000175	3.043e-15
KAF1774080.1	Ankyrin repeat-containing domain	8756	0,9	0,4	1,19	5,17	1,29	10,01	16,91	2,38	2,56	2,69	1,51	0,00502	1.378e-06
KAF1789581.1	Metallopeptidase, catalytic domain	1235	1,08	0,96	0,75	0,04	0,21	1,23	2,46	1,74	2,61	2,28	1,50	0,007432	2.231e-05
KAF1788825.1	Ankyrin repeat-containing domain	1174	34,09	38,83	36,01	33,31	39,71	0	0	0,25	0,29	0,24	-3,70	0	3.381e-24
KAF1791464.1	WD40/YVTN repeat-like-containing domain	5799	5,27	4,96	5,47	4,56	5,41	0,36	0,04	0,3	0,5	0,45	-3,24	0,000041	7.109e-35
KAF1788826.1	hypothetical protein GQ600_3050	762	2,73	1,96	1,94	0,49	1,7	0	0	0	0	0	-3,21	0,000421	1.598e-18
KAF1775491.1	WD40-repeat-containing domain	6238	2,77	2,2	2,6	2,52	1,58	0,11	0,38	0,1	0,04	0,08	-2,72	0,000065	1.234e-15
KAF1791150.1	hypothetical protein GQ600_24668	3162	9,79	11,8	12,58	13,97	13,02	3,08	1,54	0,69	0,76	0,85	-2,69	0,000062	5.411e-25
KAF1789612.1	putative domain, di-copper centre	1712	15,59	12,76	13,94	5,33	4,95	0,98	0,38	2,28	1,89	1,83	-2,54	0,000029	1.779e-39
KAF1790519.1	putative domain, di-copper centre	444	2,8	2,64	3,35	1,18	0,75	0,22	0	0,3	0,13	0,28	-2,45	0,000347	8.409e-14
KAF1789787.1	hypothetical protein GQ600_24324	311	4,45	3,72	3,79	2	1,04	0,25	0,21	0,5	0,63	0,16	-2,34	0,000161	1.435e-14
KAF1792605.1	hypothetical protein GQ600_23700	6403	11,51	13,08	11,21	29,07	23,2	4,5	3,42	2,33	2,4	1,91	-2,32	0,000008	5.930e-41
KAF1795150.1	Tetratricopeptide repeat	7222	16,15	15,16	16,02	30,86	24,7	4,68	4,97	3,57	2,48	2,61	-2,31	0,000001	1.990e-58
KAF1782989.1	WD repeat-containing protein 90	7515	5,05	4,92	5,47	5,13	4,32	1,2	1,13	0,45	0,67	0,28	-2,29	0,000164	1.408e-17
KAF1782797.1**	Thrombospondin type-1 (TSP1) repeat	7184	67,62	56,07	58,42	14,66	11,35	1,99	2,46	12,74	11,94	9,53	-2,29	0,000001	1.798e-62
KAF1783174.1	hypothetical protein GQ600_25520	558	0,78	0,84	0,88	0,41	0,71	0	0	0	0,04	0,04	-2,28	0,000328	2.793e-09
KAF1775375.1**	Carbonic anhydrase, alpha-class, conserved site	661	24,45	20,76	23,17	1,55	1,75	0,51	0,75	1,74	2,77	1,83	-2,24	0,000232	1.057e-15
KAF1793614.1	hypothetical protein GQ600_19168	3971	9,38	9,6	9,58	10,42	13,06	1,81	1,34	2,58	1,85	1,55	-2,23	0,00003	3.717e-29
KAF1783504.1	WD40-repeat-containing domain	3039	1,16	2,12	1,72	1,59	2,49	0,33	0,38	0,2	0,13	0	-2,21	0,000635	1.459e-10
KAF1783853.1	Katanin p60 subunit A1	2091	11,85	15,64	14,21	12,54	14,97	3,44	3,42	1,98	1,64	2,48	-2,19	0,000019	3.637e-31
KAF1793060.1	EF-Hand 1, calcium-binding site	804	1,46	1,92	1,85	1,26	0,62	0,04	0,13	0,1	0,17	0,33	-2,18	0,000247	3.848e-10
KAF1772692.1	EF-hand domain pair	2739	1,31	1,2	1,94	1,34	0,83	0,15	0,13	0	0	0	-2,12	0,000757	5.660e-08
KAF1789078.1	WD40-repeat-containing domain	2961	2,8	3,76	3,22	3,91	4,12	0,8	1,04	0,25	0,38	0,69	-2,12	0,000098	7.697e-17
KAF1794169.1	Organic solute carrier protein 1	5303	5,53	6,96	6,35	11,81	10,35	3,37	3,46	0,79	0,42	0,28	-2,11	0,000655	1.168e-12
KAF1782147.1	P-loop containing nucleoside triphosphate hydrolase	13632	6,65	5,24	4,85	7,94	8,48	1,34	2	1,69	0,63	0,69	-2,10	0,000126	8.209e-21
KAF1788166.1	hypothetical protein GQ600_1662	1674	0,52	0,6	0,26	0,9	0,5	0	0	0	0	0	-2,04	0,002536	2.247e-07
KAF1790794.1	Ricin B, lectin domain	2773	1,91	1,76	1,72	2,65	2,16	0,44	0,46	0,15	0,38	0,37	-1,99	0.206461	2.539e-12
KAF1791412.1	hypothetical protein GQ600_969	321	3,4	2,52	3,13	7	6,4	2,36	1,34	0,35	0,25	0,24	-1,99	0.00068388	6.132e-11
KAF1775053.1	Ricin B, lectin domain	5530	35,29	30,11	28,2	6,56	7,48	2,47	1,92	5,3	6,22	4,4	-1,98	2.95208e-05	5.305e-24
KAF1787863.1	Major facilitator superfamily domain	2128	1,68	1,28	0,97	0,29	0,91	0,07	0,08	0,1	0,13	0,12	-1,97	0.135942	1.431e-07
KAF1783713.1	WD40 repeat, conserved site	1028	1,72	1,12	1,68	3,58	2,29	0,29	0,38	0,45	0,17	0,37	-1,97	0.0804786	6.110e-10
KAF1794153.1	Enolase C-terminal domain-like	1725	2,69	2,84	2,6	8,71	12,23	1,67	1,46	0,55	0,59	1,02	-1,95	0.000144403	1.853e-15
KAF1784586.1	Immunoglobulin-like fold	1788	10,95	10,96	10,41	22,23	17,76	6,64	5,05	1,69	1,51	2,24	-1,94	0.000193322	5.483e-21
KAF1789763.1	YHYH domain	15168	9,72	8,64	8,78	34,45	32,48	7,76	5,51	2,68	1,85	2,36	-1,92	2.76201e-06	1.269e-26
KAF1793045.1	Cadherin-like	27642	25,19	23,71	24,93	66,94	58,17	6,6	2,88	7,09	6,73	3,58	-1,91	0.0907047	2.120e-08
KAF1793065.1	Tetraspanin/Peripherin	1068	0,78	0,44	0,4	1,18	1,08	0,07	0	0,1	0,08	0,04	-1,90	0.385762	1.034e-06
KAF1789775.1	Leucine-rich repeat domain, L domain-like	3068	23,7	24,51	24,58	34,57	34,31	10,77	9,14	5,16	4,92	4,97	-1,90	0.000284202	2.895e-38
KAF1775645.1	MORN motif	786	0,75	1,64	0,53	1,38	0,54	0,07	0,21	0	0,04	0,08	-1,88	0.589454	1.657e-06
KAF1794577.1	EGF-like domain, extracellular	1743	5,08	4,6	5,25	2,97	2,29	0,76	0,83	0,89	0,8	0,98	-1,88	0.154222	5.930e-16
KAF1795122.1	hypothetical protein GQ600_5281	2214	2,8	1,6	1,9	7,45	2,62	0,69	0,71	0,64	0,38	0,45	-1,84	0.0261709	7.621e-09

KAF1778784.1	Acyltransferase ChoActase/COT/CPT	2080	177,15	154,97	166,53	139,04	39,38	15,59	28,97	35,2	39,73	36,92	-1,81	0.036186	5.327e-14
KAF1789236.1	hypothetical protein GQ600_10648	1418	7,21	5,64	5,21	0,77	0,96	0,36	0,04	1,44	0,8	1,42	-1,81	0.000108385	7.288e-11
KAF1775095.1	Major facilitator superfamily domain	1774	17,01	16,6	17,03	5,33	5,9	0,47	0,42	6,64	5,21	5,37	-1,78	0.000127684	1.099e-10
KAF1787086.1	Leucine-rich repeat domain, L domain-like	768	0,49	0,64	0,4	0,49	0,42	0	0,04	0,05	0	0,04	-1,77	0.878756	9.478e-06
KAF1774875.1	hypothetical protein GQ600_17919	705	2,84	2,48	2,56	2,28	1,91	0,29	0,13	0,74	0,71	0,9	-1,75	0.0732103	4.381e-09
KAF1780886.1	Zinc finger, C2H2	3580	6,43	5,88	6,88	12,62	10,94	3,48	4,22	0,89	1,47	1,59	-1,75	0.000260975	8.806e-20
KAF1775423.1	hypothetical protein GQ600_13315	2556	0,93	1,56	0,66	1,59	2,45	0,51	0,13	0,2	0,21	0,08	-1,73	0.0662661	2.327e-06
KAF1785015.1	hypothetical protein GQ600_26539	2417	1,87	1,6	1,81	1,79	2,12	0,36	0,58	0,25	0,38	0,53	-1,71	0.603209	2.301e-09
KAF1784909.1	Nephrocystin-4	912	4,07	3,64	3,71	1,87	2,49	0,65	1,17	0,84	0,38	0,41	-1,70	0.818245	2.020e-08
KAF1779294.1	hypothetical protein GQ600_3066	831	0,49	0,52	0,66	0,77	0,71	0	0	0	0	0,16	-1,69	0.936428	2.652e-05
KAF1784585.1	Immunoglobulin-like fold	9052	10,54	12,36	10,59	70,11	63,5	20,2	16,99	2,78	2,99	3,5	-1,68	1.47137e-08	1.267e-29
KAF1793541.1	Intraflagellar transport protein 43	934	1,35	1,2	1,63	0,98	0,62	0,25	0,08	0,3	0,25	0,16	-1,68	0.264498	1.765e-06
KAF1774902.1	P-loop containing nucleoside triphosphate hydrolase	5825	0,45	0,44	0,75	3,01	2,29	0,58	0,63	0,15	0,04	0	-1,67	0.000148974	3.362e-06
KAF1780466.1	P-loop containing nucleoside triphosphate hydrolase	950	2,95	3,48	2,16	2,12	1,25	0,29	0,29	0,5	0,8	0,98	-1,66	0.0212304	2.174e-08
KAF1773637.1	Leucine-rich repeat domain, L domain-like	1404	10,47	9,84	9,4	9,41	15,3	3,37	3,8	2,08	2,73	3,13	-1,66	0.14535	3.271e-21
KAF1781432.1	Tctex-1	544	8,37	8,68	8,96	5,82	7,24	1,85	3,55	1,83	1,64	1,79	-1,66	0.790901	7.937e-15
KAF1784110.1	WD40-repeat-containing domain	4389	8,71	7,4	8,16	10,99	8,32	2,97	2,88	2,18	2,48	1,75	-1,65	0.202616	1.449e-20
KAF1781812.1	hypothetical protein GQ600_19402	768	5,87	7,56	5,82	7,65	4,41	1,63	2,88	1,19	1,05	1,71	-1,64	0.341503	2.206e-11
KAF1795309.1	Ciliary BBSome complex subunit 2, C-terminal domain	2012	5,72	6,28	4,94	3,3	2,74	0,65	0,83	1,83	1,89	1,22	-1,63	0.00655921	1.749e-14
KAF1787171.1	Acyl-CoA N-acyltransferase	468	0,86	1,64	0,84	6,8	13,81	3,23	3,01	0,1	0,17	0,16	-1,62	2.37511e-06	4.594e-07
KAF1776868.1	MORN motif	2485	0,19	0,32	0,62	0,73	0,25	0	0	0	0	0	-1,62	0.762235	4.781e-05
KAF1775975.1	S-adenosyl-L-methionine-dependent methyltransferase	808	5,91	7,2	5,34	4,19	2,87	1,23	1,84	0,89	1,14	1,42	-1,61	0.631741	3.563e-10
KAF1795857.1	Zinc/iron permease	981	7,4	6,48	6,57	28,54	22,83	2,36	2,63	2,73	3,28	2,93	-1,61	0.0296208	5.111e-08
KAF1783359.1	EGF-like domain, extracellular	683	10,47	8,64	8,78	2,36	2,04	1,2	0,79	2,08	1,85	1,59	-1,60	0.00126877	1.665e-10
KAF1788186.1	hypothetical protein GQ600_19902	315	1,87	2,04	3,18	1,87	2,99	0,58	0,58	0,69	0,46	0,69	-1,60	0.92323	1.710e-08
KAF1781982.1	Concanavalin A-like lectin/glucanase domain	2343	23,66	20,88	21,4	27,24	31,69	9,46	16,03	3,92	4,79	5,62	-1,60	0.00101148	2.475e-17
KAF1794122.1	Intra-flagellar transport protein 57	1354	4,75	4,36	4,77	1,83	2,12	0,87	0,88	0,89	0,97	0,77	-1,60	0.0911234	6.654e-10
KAF1781231.1	hypothetical protein GQ600_5796	12398	5,61	4,08	4,72	2,85	2,33	0,83	0,42	1,39	1,35	1,47	-1,58	0.00930223	2.385e-11
KAF1778945.1	Ankyrin repeat-containing domain	1143	7,63	7,64	6,53	8,96	6,45	1,45	3,92	1,83	1,35	2,24	-1,58	0.337256	3.563e-12
KAF1787239.1	P-loop containing nucleoside triphosphate hydrolase	449	1,31	2,32	1,77	1,18	0,08	0,04	0	0	0,04	0,41	-1,56	0.123409	1.293e-04
KAF1794774.1	Sec8 exocyst complex component specific domain	3016	21,9	22,36	24,98	20,19	22,91	8,12	7,39	6,44	6,1	6,55	-1,56	0.515297	2.650e-29
KAF1772290.1	Cyclic nucleotide-binding-like	2942	2,17	2,32	1,72	2,4	3,24	0,69	0,13	0,79	0,63	0,45	-1,56	0.787437	2.966e-06
KAF1793047.1	hypothetical protein GQ600_3185	5764	150,98	139,45	130,88	115,31	153,98	25,82	26,68	55,97	55,71	53,41	-1,56	0.0460971	5.665e-18
KAF1781408.1	P-loop containing nucleoside triphosphate hydrolase	4018	40,11	36,39	34,73	73	139,8	35,24	29,26	10,31	12,28	10,42	-1,55	6.99263e-06	2.798e-18
KAF1781229.1	hypothetical protein GQ600_5794	954	1,01	1,08	1,19	0,98	0,96	0,22	0	0,3	0,38	0,12	-1,54	0.537026	4.005e-05
KAF1786000.1	EF-Hand 1, calcium-binding site	939	3,92	5,08	5,12	0,49	1	0,25	0	1,29	1,35	1,38	-1,54	0.000129071	7.231e-08
KAF1772261.1	Major facilitator superfamily domain	1248	0,64	0,52	0,22	1,18	0,83	0,18	0,21	0,05	0,08	0	-1,53	0.0405746	1.335e-04
KAF1773437.1	P-loop containing nucleoside triphosphate hydrolase	613	5,94	6,56	5,6	3,46	2,04	1,05	0,63	1,34	2,27	0,94	-1,53	0.0267777	2.020e-08

KAF1773201.1	GAF domain-like	3286	14,28	12,8	12,93	9,81	8,9	2,07	4,17	4,36	4,46	3,38	-1,53	0.125763	2.908e-22
KAF1790437.1	IQ motif, EF-hand binding site	2104	0,93	0,72	0,35	1,38	1,7	0,47	0,08	0,1	0,08	0,12	-1,52	0.0311586	9.994e-05
KAF1784324.1	Galactose-binding domain-like	6319	0,71	1,12	1,5	0,9	0,42	0,04	0,29	0,05	0,21	0,2	-1,51	0.517091	1.300e-04
KAF1774614.1	hypothetical protein GQ600_16164	351	1,35	1,72	1,41	0,81	0,33	0,29	0	0,25	0,04	0,16	-1,51	0.305888	1.918e-04
KAF1793084.1	hypothetical protein GQ600_12977	1566	0,3	0,48	0,35	0,86	0,33	0,04	0	0,1	0	0,04	-1,51	0.802848	2.581e-04
KAF1781052.1	ADP-ribosylation factor-like 2-binding protein, domain	625	0,93	0,92	0,93	0,37	0,62	0,07	0,21	0,05	0,21	0,12	-1,51	0,0043	9.823e-05
KAF1779880.1	hypothetical protein GQ600_7175	1449	7,36	7,04	7,94	4,28	4,49	0,94	2,71	0,89	2,44	1,63	-1,50	0,002352	2.896e-08
KAF1778883.1	P-loop containing nucleoside triphosphate hydrolase	3042	1,76	1,4	1,24	5,74	7,07	1,81	0,54	0,25	0,42	0,65	-1,50	0,004523	5.704e-06

Supplementary Table S1. The 120 differentially expressed genes in the virus free PhF66– and PhF66 containing two bunyaviruses with cutoff values > 1,5 revealed by RNA-seq. *=proteins also found differentially expressed in the shotgun proteomic analysis. **=proteins detected in shotgun proteomic analysis, but not differentially abundant.
