# Supplementary figures and images for: Bunyaviruses Affect Growth, Sporulation, and Elicitin Production in Phytophthora cactorum
Source: Viruses. 2022 Nov 22;14(12):2596. doi: 10.3390/v14122596 (PMC9788385; doi:10.3390/v14122596)

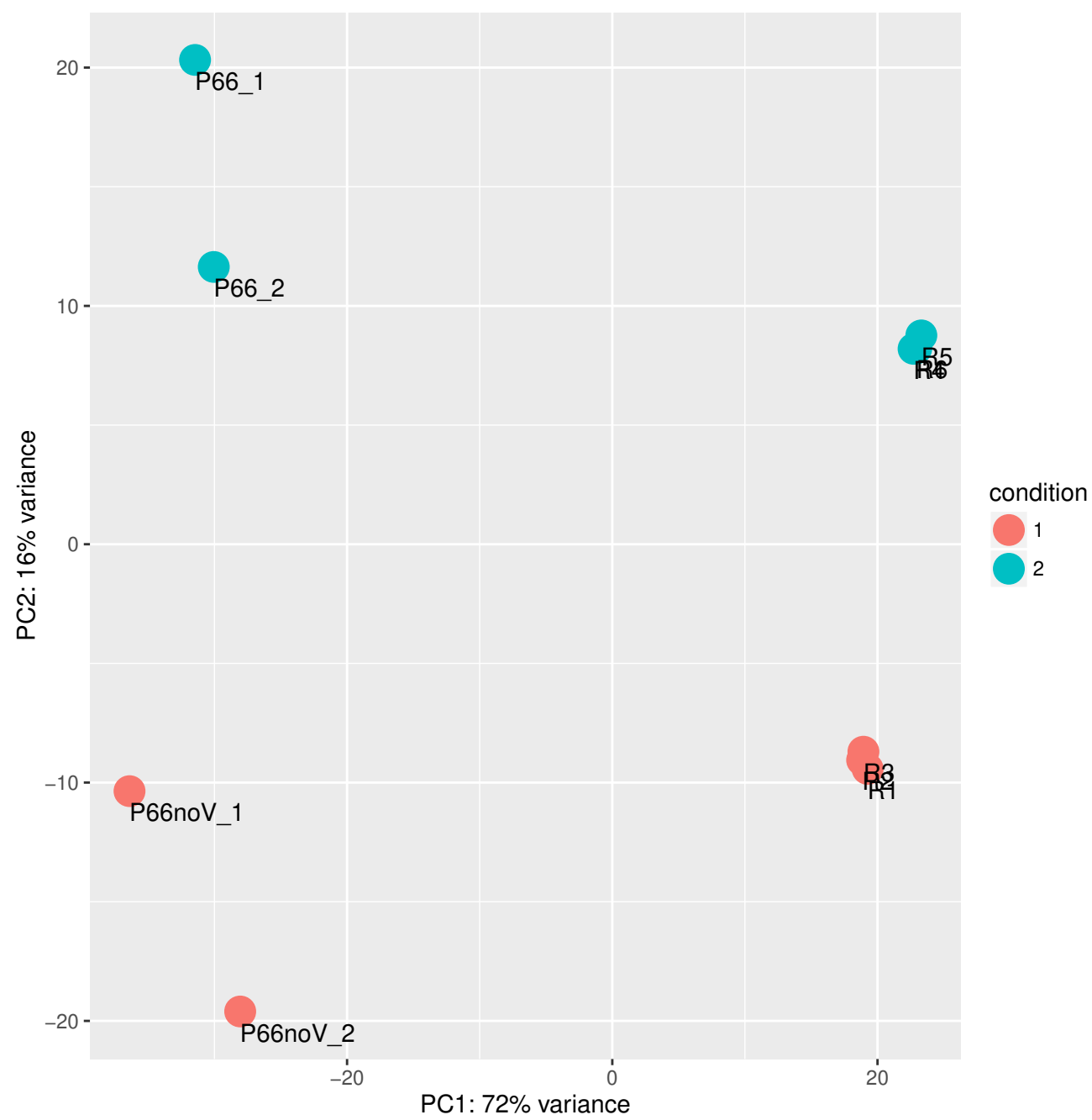

Color Key  
and Histogram

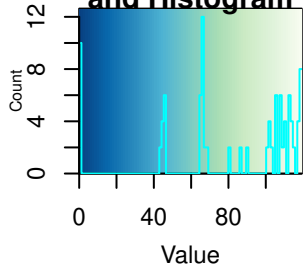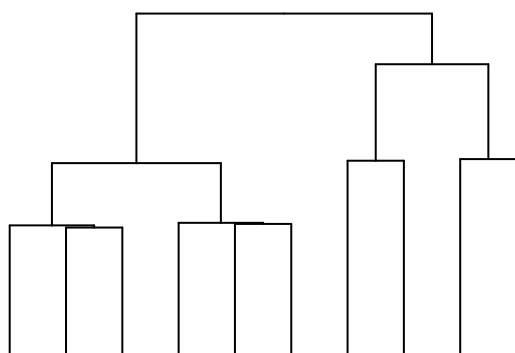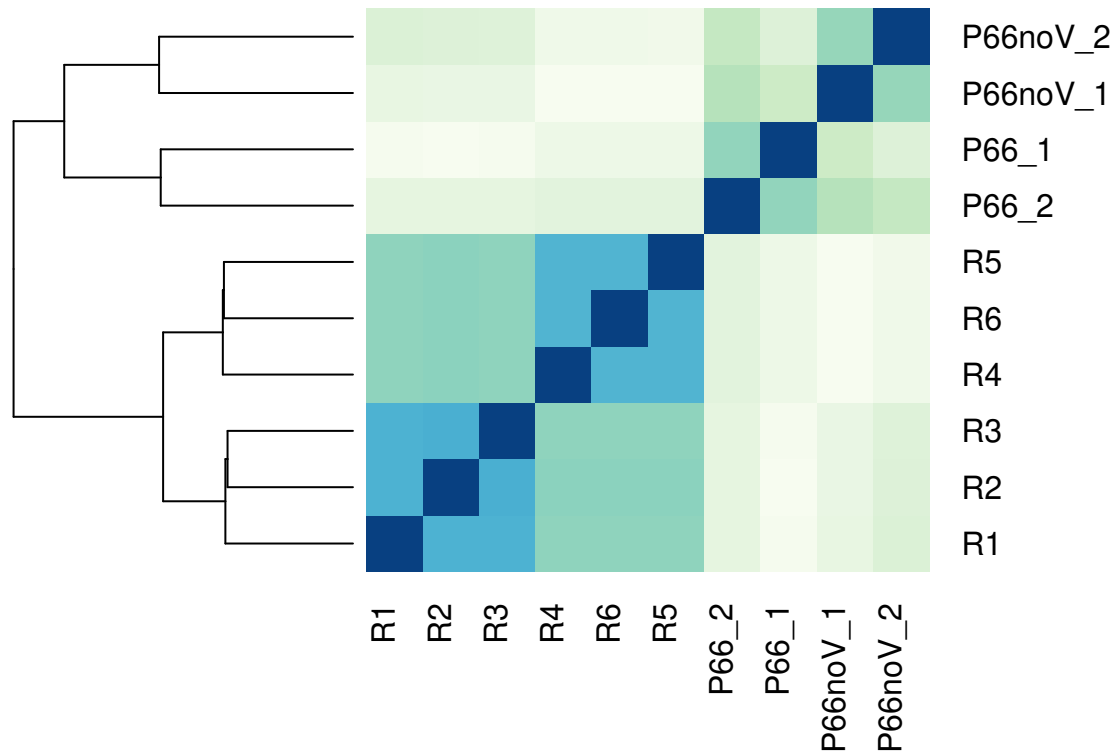

Supplement: Supplementary file 1 [file viruses-14-02596-s001.zip › Supplementary1_PCA_and_heatmap_deseq2.pdf]
